# The Design and Implementation of the Leaf Area Index Sensor

**DOI:** 10.3390/s150306250

**Published:** 2015-03-13

**Authors:** Xiuhong Li, Qiang Liu, Rongjin Yang, Haijing Zhang, Jialin Zhang, Erli Cai

**Affiliations:** 1 College of Global Change and Earth System Science, Beijing Normal University, No.19, XinJieKou Wai Street, HaiDian District, Beijing 100875, China; E-Mails: lixh@bnu.edu.cn (X.L.); toliuqiang@bnu.edu.cn (Q.L.); hjzhang89@126.com (H.Z.); llbsh123@163.com (J.Z.); celcugb@163.com (E.C.); 2 Joint Center for Global Change Studies, Beijing 100875, China; 3 State Key Laboratory of Remote Sensing Science, Jointly Sponsored by the Institute of Remote Sensing and Digital Earth of Chinese Academy of Sciences and Beijing Normal University, No. 20 Nouth, DaTun Road, ChaoYang District, Beijing 100101, China; 4 Chinese Research Academy of Environment Sciences, No.8, DaYangFang, AnWai, ChaoYang District, Beijing 100012, China

**Keywords:** leaf area index, wireless sensor network, remote upgrade, validation

## Abstract

The quick and accurate acquisition of crop growth parameters on a large scale is important for agricultural management and food security. The combination of photographic and wireless sensor network (WSN) techniques can be used to collect agricultural information, such as leaf area index (LAI), over long distances and in real time. Such acquisition not only provides farmers with photographs of crops and suggestions for farmland management, but also the collected quantitative parameters, such as LAI, can be used to support large scale research in ecology, hydrology, remote sensing, *etc*. The present research developed a Leaf Area Index Sensor (LAIS) to continuously monitor the growth of crops in several sampling points, and applied 3G/WIFI communication technology to remotely collect (and remotely setup and upgrade) crop photos in real-time. Then the crop photos are automatically processed and LAI is estimated based on the improved leaf area index of Lang and Xiang (LAILX) algorithm in LAIS. The research also constructed a database of images and other information relating to crop management. The leaf length and width method (LAILLW) can accurately measure LAI through direct field harvest. The LAIS has been tested in several exemplary applications, and validation with LAI from LAILLW. The LAI acquired by LAIS had been proved reliable.

## 1. Introduction

The quick and accurate acquisition of parameters related to agricultural conditions on a large scale has been emphasized by agriculture management, crop yield prediction, as well as ecological research [1]. The accuracy of regional farmland parameter monitoring is relatively weak, which is one of the bottlenecks of the remote sensor monitoring of agricultural conditions. Leaf Area Index (LAI) is a typical regional farmland parameter that is closely related to the final production of crops and to the effectiveness of agricultural production. The current routine method involves the monitoring of the LAI by remote sensing retrieval. However, the monitoring of LAI is strongly influenced by time, season and space, and the different sensors used. Scales or retrieval algorithm can lead to distinct differences in LAI remote sensing monitoring results. Thus, examining the quality of these products and improving their accuracies in relation to ground measurement data have become urgent for current eco-agricultural remote sensing applications [2]. Ground measurement has many problems, such as being labor- and time-consuming, lacking in spatial representativeness and being discontinuous, while the advanced technology of wireless sensor nets provides exactly the means to acquire vast amounts of ground measurement data at a low cost. Therefore, it is necessary to apply quantitative remote sensing technologies, and ground sensor nets technologies, to improve the accuracy and efficiency of farmland monitoring.

Ground LAI measurements are generally carried out in two ways. The first is direct measurement, which involves the collecting of leaves by harvesting, destructive sampling, litter collection *etc.*, and acquires the leaf areas with scanning planimeter (e.g., Li-3100, LI-COR, Lincoln, NE, USA; CI-202, CID Inc., NW Camas, WA, USA), or gravimetric method, or based on the relations between the leaf sizes and leaf areas that are determined by shape factors [3]. The direct measurement of LAI leads to relatively reliable results, but is usually highly destructive or involves large amount of manual work. It is impractical to use direct measurement in research and applications in large region or demanding crop dynamics because of this high cost and disturbance to crop growth. The leaf length and width method (LAILLW) is one of them. This method is precise and often used for validation of other LAI measured methods. The second way is indirect measurement, in which the LAI is calculated based on radiation transfer theory and measurements of light in different height of canopy [4,5]. The indirect measurements are non-destructive and the labor costs are greatly reduced; but the price of concerned instruments may be higher. The most frequently used indirect LAI measurement instruments are shortly introduced as follows. The SunSCAN (Delta-T Devices Ltd., Cambridge, UK) and the AccuPAR (Decagon Devices, Pullman, WA, USA) can be used under direct solar radiation and at wavelengths of the photosynthetic active radiation (PAR). It measures the fraction of PAR absorbed by canopy and also exports LAI as an auxiliary product. The LAI-2000 (LI-COR, Lincoln, NE, USA) and its successors are fish-eye light sensors, and by measuring the light in several ranges of zenith angles both at the top and bottom of canopy, thus derive the directional transmittance of canopy and thereby the effective LAI. These instruments have to repeat the measurement several times before making calculations, and the measurements is recommended to be arranged in ambient light conditions to void the influence of strong sun light. The Tracing Radiation and Architecture of Canopies (TRAC) [6] is designed mainly for tree canopy and take considerations on the clustering effects of leaves. A major output of TRAC is the clumping index (CI) which converts the effective LAI into true LAI. All the advantage and disadvantages of these methods have been extensively reviewed in [7]. The Leaf Area Index Sensor (LAIS) is a new integrative indirect measurement method using WSN technology.

A branch of indirect LAI measurement methods make use of digital camera photographs, for example, the Multiband Vegetation Imager (MVI) [8]. The advantage of digital photography is that photos contain large amounts of information, e.g., color, shape, that can be extracted by suitable image processing software, according to the development of algorithms, so these image processing algorithms are an important aspect of the LAI sensor. The crop canopy is not as high as trees, thus downward looking cameras should be chosen for crop monitoring, but studies on downward looking canopy photo algorithms appeared later than those of upward photos [9,10], and the influence of the complex background must be considered. When indirect measurements are employed to measure LAI, systematic errors may occur due to the uncertainties in the interference factors, such as leaf angles and clumping index, so it is critical to validate the accuracy of the results of indirect LAI measurements, and perform calibrations, if necessary, by more robust direct measurement. The leaf area index of Lang and Xiang (LAILX) is a mature and stable method of obtaining the LAI from the digital photographs. The LAILX used in LAIS had been improved.

Currently all the above LAI measurement methods and instruments are operated by humans and cannot work automatically in the field. With the rapid development of space technology, the dynamics of crop growth have been monitored by remote sensing, but is hard to capture in the validation sites. An efficient and economical way to measure LAI in real-time is urgently needed. Based on these demands, the authors combined ground-based monitoring technology with a wireless sensor network to monitor LAI and developed the LAIS, which can achieve real-time, automatic and continuous monitoring [11,12]. The combination of LAIS’s fixed-point *in situ* measurement and remote sensing LAI inversion technology will help provide more efficient and precise monitoring of the dynamics of crop growth on the large scale. The LAI from LAIS had been validated by the LAI from LAILLW.

## 2. System Design

### 2.1. System Introduction

**Figure 1:**
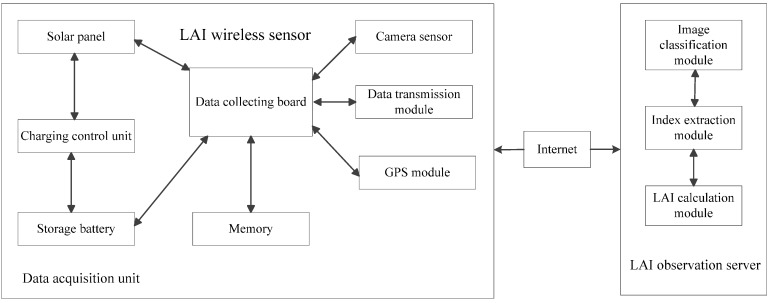
System structural framework.

This LAIS system is composed of hardware and software parts (Figure 1). The hardware system (or LAI sensor node) collects digital photos of plant canopies and transmits them to the LAIS data server. The software system helps to interpret the photos of plant canopies and automatically extracts LAI, and the batch processing function of this software makes it convenient for users to deal with the large quantities of data generated.

### 2.2. The Hardware of the LAIS

**Figure 2:**
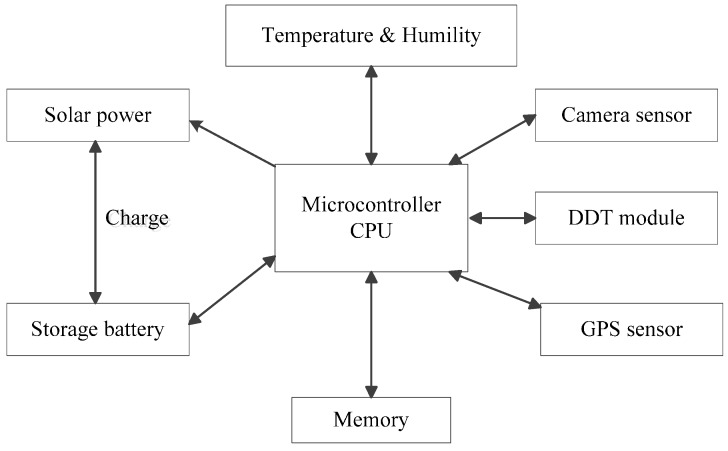
Hardware components of the LAIS.

The hardware of the LAI wireless sensor includes four modules: a microcontroller CPU, a sensor module, data transmission and smart power management systems. Figure 2 illustrates the hardware components. There is a Camera Sensor, a GPS Sensor and a Temperature Humidity Sensor in the sensor module. Users can expand the sensors according to their demands. The Data Transmission Terminal (DTT) can be linked with a 2.4 G Module, an Iridium Data Module, a Radio Module, a 3 G Module and a WIFI Module. The microcontroller CPU with the embedded ZKOS operating system manages the whole system.

In the smart solar power generation system, electronic power comes from the solar panel whose upper limit is 19 V. The solar power will automatically supply power when its voltage is higher than that of the battery. When solar power is sufficient, the sun supplies power to the system; when the solar power is insufficient, the battery supplies power to the system. When the voltage of the battery is below 10 V, the system will hibernate automatically to protect the battery life till the system is up started the next time.

The Temperature Humidity Sensor adopts the Digital Sensor sht10 with a voltage of 3.3 V. The module uses voltage from the power supply only during sampling. The Camera Sensor adopts a 5 megapixel camera with a resolution of 320 × 240–2596 × 1944 and can be set up to identify the light intensity automatically, and the flash can be started shooting when the light intensity is low. The voltage of the power supply should be 12 V to support the module’s regular work. The power is only switched on while shooting.

The DTT Module adopts a voltage of 3.8 V, which is applied after the shooting tasks are finished. The program can be modified. This module is started and logs in hourly, which guarantees the Internet connection even if the DTT network signal is weak and guarantees remote transmission of the data. Once the remote server logs in, it transmits the data in the storage, and then the module is switched off automatically.

The GPS Sensor uses a voltage of 5 V, starts at 12 o’clock midnight every day, and automatically adjusts the RTC real-time clock and records the coordinates. The GPS Sensor is off the remainder of the time. The storage uses a 1 G SD card. The data on the card is automatically deleted after being read to create space for new data.

### 2.3. The Software of the LAIS

#### 2.3.1. Embedded Operating System: ZKOS in the CPU

**Figure 3:**
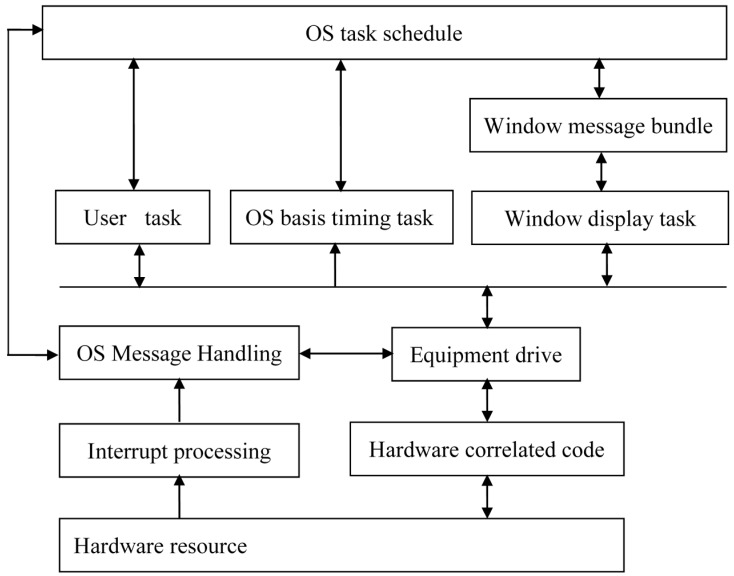
The software structure of ZKOS.

The embedded operating system features minimal code, reduced dependence on stacks, registers, timers and interrupters and is applicable to different single chips [13]. The software structure of the ZKOS embedded operating system is shown in Figure 3.

**Figure 4:**
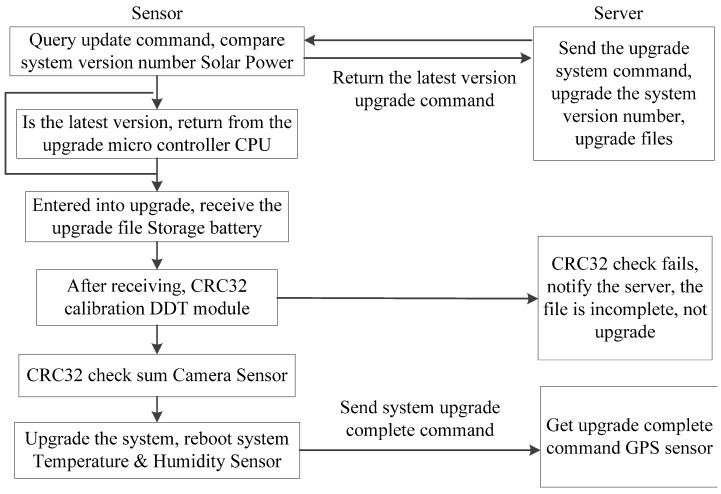
Flow chart of the remote upgrade task.

The ZKOS embedded operating system controls the operation of the entire system, which initiates every task at a set time:

(1) The task of photographing: The shooting time can be set by the user, and this task can be completed a maximum of 12 times per day. The system takes photos according to the set time and uploads the results to the SD card.
(2) The task of the Data Transmission Terminal (DTT) module: This task is initiated after the photographing task is completed. Following remote logins, the data in the storage, which includes the collected crop photos, temperature, humidity and system voltage, are transmitted so that the server acknowledges the system operation conditions.
(3) The task of the GPS module: This task is initiated at 12 o’clock midnight (a default system setting that can be reset) and searches for satellites. When a satellite is found, the GPS module modifies the time for the system RTC module, records the longitude and latitude of the system and sends this information to the remote server along with the crop photo.
(4) The task of remote upgrades: During system operation, it may be necessary to upgrade the software to solve specific problems. This task can be accomplished by remotely downloading programs from server. Because going to the field can be difficult in some circumstances, the system uses communication networks to achieve remote upgrades, as the communication networks require no site wiring, maintain real-time online connections, charge by the byte, and provide quick log ins and high-speed transmission. The system divides the space of the FLASH storage in the microcontroller CPU, defines the data format of the upgrade file transmission, and then accomplishes the system software’s remote upgrade via FTP technology. The specific procedure is shown in Figure 4.

#### 2.3.2. The Software in the LAI Server

##### Workflow

**Figure 5:**
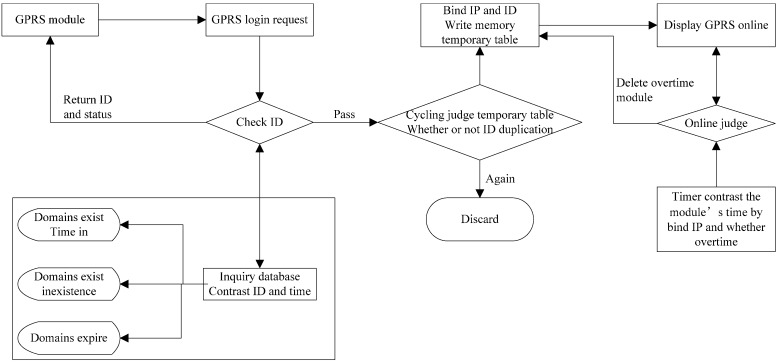
The workflow of the LAIS server.

The LAIS server is located in Beijing and remains open to monitors the terminal signals all day. When an online order is received, the ID of the module is checked for existence, module expiration, and module ID, and whether the address and the IP address conflict with each other is checked. If all of these checks are verified, the module is added to the online module group and the IP address is stored. If something goes wrong, a malfunction code is returned to the server. If the data transfer terminal (DTT) maintaining message is received, the time of the online module will be updated to the last communication time. The overtime module or the offline module and all connected resources are cleaned up after the timer traverses the online list according to the set time. The server finds the project for the module and the DLL file that needs to be analyzed after receiving the data. The server then dynamically calls the DLL, analyzes the data and stores the data in the database. The work flow of the data center is shown in Figure 5.

##### Data Transmission

**Figure 6:**
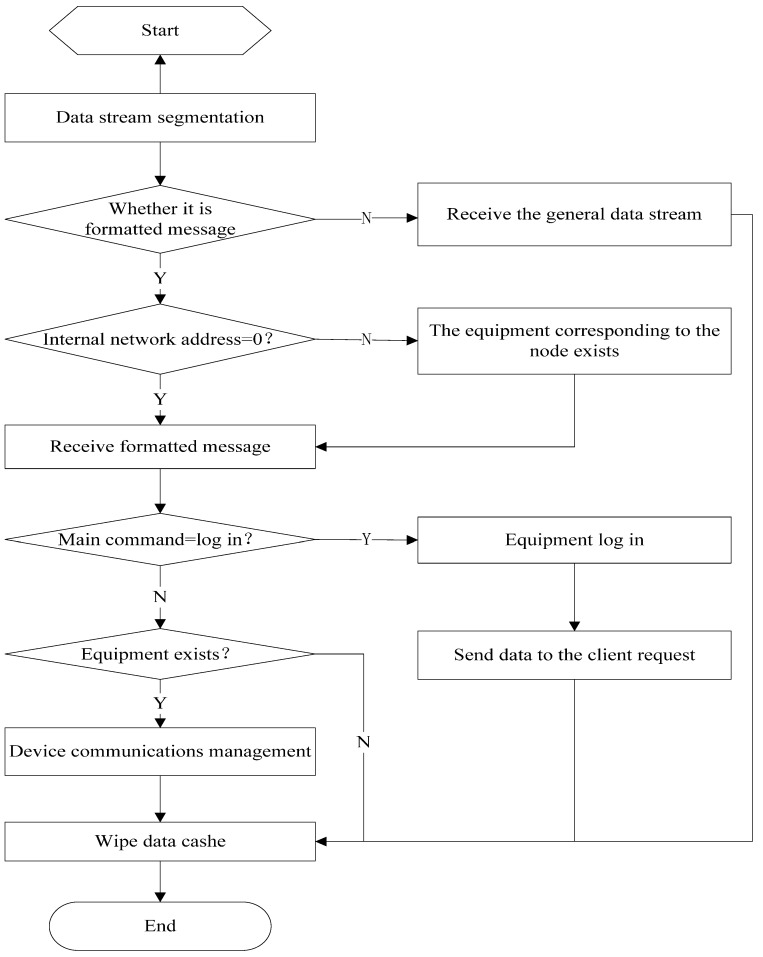
Data reception by the server.

After connecting with the server, the client sends the data to the server. The server receives the data frames and divides the frames with the Data Flow Partition Function to confirm the formatted message data. The function confirms the data sources, acquires the client’s IP address and the positions of the frame header and footer, and confirms the data type between the server and the message data; thus, the server can judge whether the data are normal flow data or formatted message data, and then saves the respective data into separate byte array types for subsequent processing. Next, the intranet address field in the formatted message is checked to determine whether the client is a base station or a node in the wireless sensor network. If the main command is a login command, the client logs in. Otherwise, the server object begins communication management with the respective client. The specific work procedure is shown in Figure 6.

**Figure 7:**
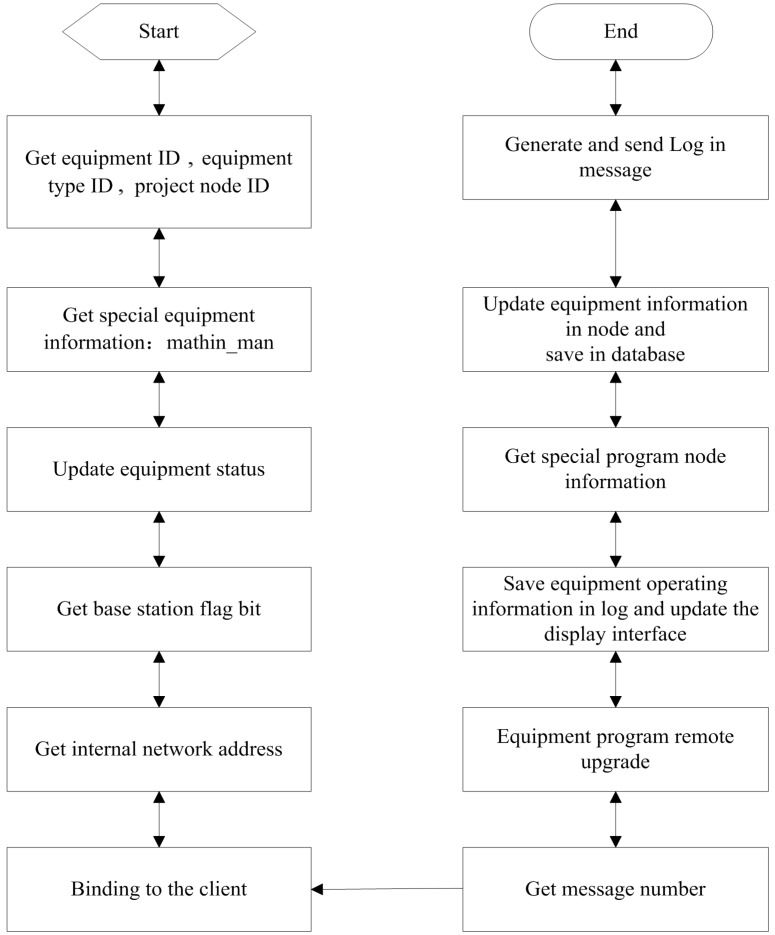
Data transmission during the login process.

The client sends the formatted message with the login command information. After reception, the server acquires the relative login information and then finishes the device login. First, the server acquires the specific information of the client login device through its ID and the ID of the device type. Next, the server binds the client with the information of the current device management and then acquires the version number of the program in the device login message and the field information of the upgrade starting address to accomplish the device program’s upgrade management. Finally, the server confirms the information of the program’s nodes, which corresponds to the client device, upgrades the information of the corresponding devices in the nodes and save that information to the database. Thus far, the device has logged in successfully. During this process, the program records its relative device operations in the journals and displays the upgrade on the interface. The specific working procedure of the client’s login is shown in Figure 7.

##### Image Parsing

Using dynamic link-base technology in the LAIS observation server to integrating the data analysis function into the Dynamic Link Library functions to fully meeting the upper demand of development; the function can be called by the main thread. The main thread calls for different analytical DLLs to analyze data from different device types. This design pattern can greatly improve the universality and portability of the program code.

The DLL cannot work independently and can only be called by other programs. Therefore, C# program developers typically build an EXE as Server 1 and a DLL as the Analysis and are then able to call the DLL in Server 1. In this manner, both the DLL and its connections with the application programs can be debugged.

**Figure 8:**
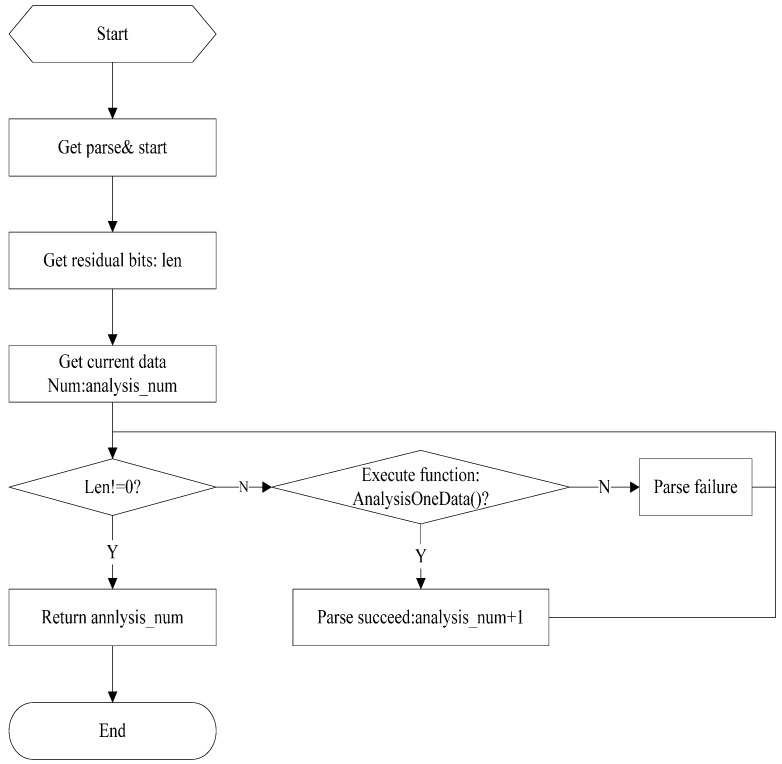
The data analysis workflow.

The data analysis program in the LAIS is a dependent module that does not change with application programs, hence can be transformed into a DLL and published with application programs. In this manner, the universality of the software is ensured, which makes it possible for the data analysis module to be maintained when new application software is developed in the future. Furthermore, programs can be developed in modules, which speeds the process of program development. Moreover, because the data analysis program and Server 1 are independent, modifications of one will not affect the other. The specific data analysis procedure is shown in Figure 8. The program automatically analyzes the image data from the client and saves it for the subsequent collection of the LAI.

## 3. LAI Estimation

### 3.1. Main Algorithm—Improved Lang and Xiang Method

The leaf area index of Lang and Xiang (LAILX) is also called the limited length average method. It is a theory that is used to indirectly measure the leaf area indexes of heterogeneous canopies. The key to this method is the counting of the gap fraction by segmenting within the entire path. This method assumes that the leaves are distributed randomly in each partition units. If each unit is equal, the actual LAI equals to the arithmetic average of the LAIs of all the partition units (paths), and the effective LAI can be calculated with the Poisson theorem [14,15,16,17,18,19]. Then, the actual LAI is derived by effective LAI divided by the clumping index (CI). The expression of clumping index of Lang and Xiang is as follows:

(1) $$\Omega {\left(\text{θ}\right)}_{LX}=\frac{L_{e}}{L_{t}}=\frac{\ln\bar{P_{0i}}}{\ln P_{oi}}=\frac{\ln\left(mean\left(P_{cell}\left(\text{θ}\right)\right)\right)}{mean\left(\ln\left({\text{P}}_{\text{cell }}\left(\text{θ}\right)\right)\right)}$$

where Ω(θ)*_LX_* is the CI, the *L_e_* is the effect LAI of the scene, *L_t_* is the true LAI of the scene, *P_cell_*(θ) is the gap fraction of every partition unit, and the average gap fraction of all partition units is mean(*P_cell_*(θ)). To introduce the improvements to LAILX method, we should go to the details of its basic assumptions.

When the leaves in the partition units are in finite size and distributed randomly, the gap fraction of the canopy can be expressed following the binomial distribution:

(2) $$P_{0}={\left(1-\frac{G}{\mu }\frac{\Delta L}{\Delta A}\right)}^{L_{i}\Delta A/\Delta l},\;\mu =\cos\left(\text{θ}\right)$$

where *P*_0_ is the gap fraction of, ΔL is the average area of single leaf, G is the phase function which is related to leaf angle distribution, ΔA is the partition unit area, *P*_0_ is the LAI of unit i, the observation angle is θ.

Accordingly, when *P*_0_ (the measured gap fraction of partition units) is known, the calculated LAI is:

(3) $$L_{i}^{ˊ}=\frac{\Delta L}{\Delta A}\frac{\ln\left(P_{0}\right)}{\ln\left(1-\frac{G}{\mu }\frac{\Delta L}{\Delta A}\right)}$$

On the other hand, if the size of the leaves in the partition units tend toward infinitesimal, the leaves match the Poisson distribution, and the LAI will be:

(4) $$L_{i}^{〞}=-\frac{\ln\left(P_{0}\right)}{G/\mu }$$

There is a problem in the application of the method of Lang and Xiang; the area of the partition unit ΔA is required to make the distribution of leaves in partition units close to a Poisson distribution. The optimal area of the partition unit ΔA is supposed to meet two conditions. First, the binomial distribution can be approximately regarded as a Poisson distribution. Second, the gap fraction of the partition units *P*_0_ is greater than 0. The calculation error of LAI of the partition units is partly due to the difference between the binomial leaves distribution and the Poisson distribution in partition units. Therefore, the area of the partition units ΔA would be calculated with error analysis. When the leaves in partition units meet the Poisson distribution, the estimated LAI $L_{i}^{〞}$ will be higher than the $L_{i}^{ˊ}$ when the leaves meet the binomial distribution. Thus, the relative error ε*_i_* is defined as the estimation of the uncertainty of the LAI in partition units:

(5) $${\text{ε}}_{i}=\frac{L_{i}^{〞}-L_{i}^{ˊ}}{L_{i}^{ˊ}}-\frac{\mu }{G}\;\frac{\Delta A}{\Delta L}\;\ln\left(1-\frac{\mu }{G}\;\frac{\Delta L}{\Delta A}\right)-1$$

Defining r as the ratio of the partition units area and the average projected area of single leaf on horizontal plane, the relative error is actually a function of r:

(6) $$r=\frac{\text{μ}}{G}\frac{\Delta A}{\Delta L}$$

(7) $$\text{ε}\left(\text{r}\right)=-r\ln\left(1-\frac{1}{r}\right)-1$$

When r = 100, ε(r) is 0.5%, which is regarded as the acceptable error range of the deviation of the Poisson distribution from the binomial distribution. Under this circumstance, the optimal partition unit area is $\Delta A=100\frac{G}{\mu }\Delta L$. The physical meaning is that the optimal area of partition unit is 100 times the average projected area of single leaf. In addition, if a partition units have a gap fraction of zero, it is identified and removed in the calculation. By applying the optimal partition units size, the improved LAILX method becomes more stable and accurate, and it can be operated automatically on the data server without human supervision.

### 3.2. Software of the LAI Estimation

**Figure 9:**
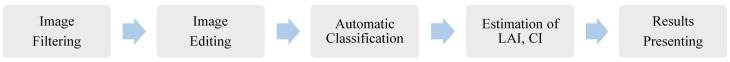
Flow of the collection of growth parameters from canopy images.

To determine the LAI from the digital photographs of the crop canopies; parameters including the LAI; gap fraction and clumping index need to be collected via the inputting and editing of digital images; automatically classifying the images into binary images; information collecting from binary images and calculating of the LAI (see Figure 9).

#### 3.2.1. Image Filter

Occasionally images cannot be processed due to the weather, abnormal decoding of image data or low gap fraction. Therefore, it is necessary to filter the data collected by the sensor and to identify images that meet the requirements, which can be correctly processed to collect accurate growth parameters.

#### 3.2.2. Image Editing

**Figure 10:**
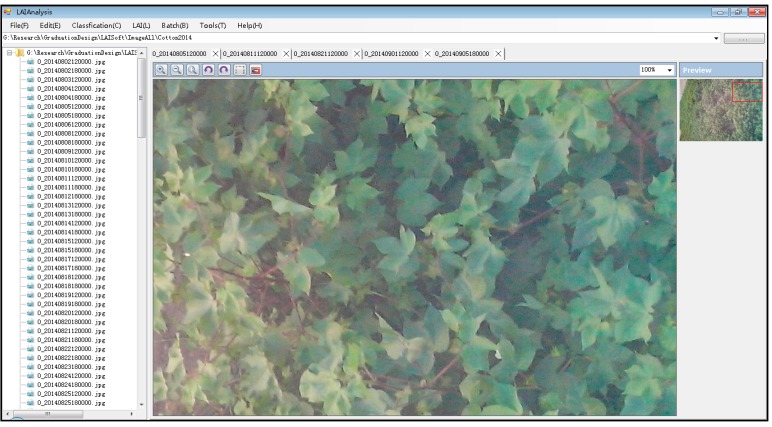
Image editing.

Image editing refers to the preprocessing of the original digital photographs. The selected images that meet the requirements can be viewed in the growth parameter collection software and input in the image processing window. The software has basic functions that can scale, rotate, restore, select and preview images. It is simple to edit images in this version of software with subsegment which were cut from the original digital photographs and can be served as the data source (see Figure 10).

#### 3.2.3. Binary Images Produced by Automatic Classification

This is a key step in the classification of the images and can be designed to automatically perform batch processing. Typically, the digital photos of the canopies of the crops are classified into soil background and green vegetation. It is suggested that various classification methods should be adopted for winter wheat and corn and even for corn in its different growth phases. This software offers three methods, including improved K-means classification, automatic threshold classification and partition threshold classification. Figure 10 shows the results when the automatic threshold classification 1 and the default parameters are adopted to classify images.

Five methods of band threshold classification for winter wheat and corn are proposed herein. R, G and B represent the red, green and blue band, respectively, in the RGB color space. H refers to the chroma in the HSL color space, and t1, t2, t3 and t4 refer to different thresholds. In each of the following five methods, the program offers recommended thresholds, and proper methods should be chosen accordingly. The methods can be switched by clicking the drop-down menu, which can change thresholds to the recommended defaults automatically, and results will be shown in the right side of the window:

Method 1: (t1 < H < t2) or (R > t3 and G > t3 and B > t3), t1 = 60, t2 = 180, t3 = 200
Method 2: (G > R + t1 and G > B + t2) or (R > t3 and G > t3 and B > t3), t1 = 0, t2 = 0, t3 = 200
Method 3: (t1 < H < t2) and (G > R + t3 and G > B + t4), t1 = 60, t2 = 180, t3 = 0, t4 = 0
Method 4: (G > R + t1 and G > B + t2) or ((R − 2G) < t3) or (R > t4 and G > t4 and B > t4), t1 = 0, t2 = 0, t3 = 10, t4 = 200
Method 5: ((G − R)/(G + R) > 0.3 + t1) or (R > t4 and G > t4 and B > t4), t1=0, t2=0, t3=0, t4=200.

#### 3.2.4. LAI and CI Estimation from Binary Images

Gap fraction and gap size can be collected from the statistics of the binary images, and then the clumping index (CI) and LAI can be estimated with the improved LAILX method. The software sets the configuration files that provide crop-specific parameters to ensure accurate and valid estimation of LAI. These crop-specific parameters include vegetation species, width of the rows, approximate leaf size, region of interest, *etc.* Among these parameters, the region of interest refer to the region in which the crop canopy is representative to the field and image geometric and radiometric distortions are small, thus avoid errors caused by obtruding objects or inaccurate classification results.

#### 3.2.5. Results Presenting

The results of the crop growth parameters are output in a text file format including the LAI and CI.

## 4. Field Measurements and Validation Experiments

### 4.1. Introduction of Field Measurements

**Figure 11:**
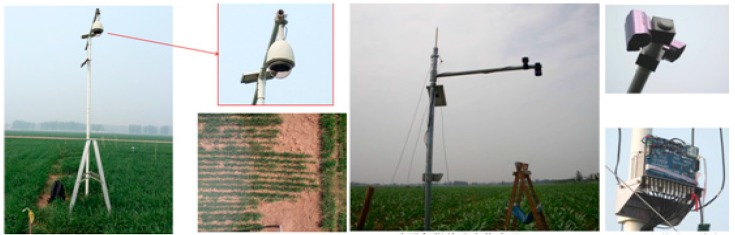
Field photos.

Since 2010, LAI field measurements have been continuously observed for corn, cotton and wheat, in Huailai of Hebei Province, the Xiaotangshan Agricultural Demonstration Base of Beijing and Hebi of Henan Province (Figure 11). LAI for corn have been measured in Liuzhai, Weixian and Wangzhuang of Junxian of Hebi in 2010 and 2014. LAI for corn, wheat and cotton have been measured at the Huailai Remote Sensing Comprehensive Test Station from 2012 to 2014. LAI for wheat have been measured ate the Xiaotangshan Agricultural Demonstration Base in 2014. In all four continuous LAI time series for corn, three continuous LAI time series for wheat, two continuous LAI time series for cotton have been obtained during the growing season of each crop.

### 4.2. Data Validation

This was a preliminary validation of the instruments and measurements. Validation experiments have been performed utilizing the direct measurement of LAI with the leaf length and width method (LAILLW), and the observed value of the LAI2200.

#### 4.2.1. Introduction of LAILLW Method

This method aims to acquire direct LAI measurement in field without too much destruction to the canopy. Here, corn is used as an example. This method involves outdoor and indoor measurements. Outdoor measurement includes three steps: counting the numbers of corn plants in unit area, measuring the length and width of every leaf on typical plants, and harvesting typical corn plants for indoor measurement. Indoor measurement primarily entails measuring the length, width and area of every leaf in every corn plant sample.

The formulas are:

(8) LAI=S×N

(9) S=f(len×wid)

In these formulas, LAI refers to the leaf area index, S refers to the area of a single plant, N refers to the number of plants in a unit area, f(len×wid) refers to the function of the leaf area and length and width of leaf.

To protect crops and reduce their destruction, the area of a single leaf was taken as the product of its length, width and a correction factor called the shape factor here; thus, this method is also called the shape factor method in some other articles. According to this method, the calculation of single leaf area is shown in Equation (10). As shown in this formula, the key to this method is how the shape factor is acquired. By measuring typical corn indoors, the length and width of every leaf are acquired, and the corresponding leaf areas are scanned by CI202. Thus, the shape factors of these typical plants are acquired, the average of these values is taken as the average shape factor of all the species:

(10) $$\text{s}=f\left(len\times wid\right)=len\times wid\times f_{l}$$

In the above formula, *len* and *wid* refer to the length and width of leaf, respectively, and *f_l_* refers to the shape factor.

#### 4.2.2. Validation of the LAI from LAIS Based on the Improved LAILX Method

**Figure 12:**
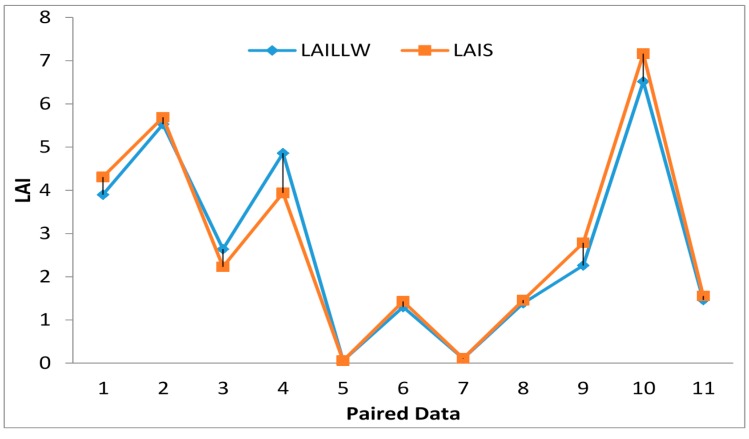
Comparison of LAI from LAILLW and LAIS.

LAILLW is a direct measurement method for LAI to serve the purpose of validating the indirect LAILX method. Most values of the LAIS based on the improved LAILX method are larger than those of the LAILLW and also close to the LAILLW (Figure 12) because the LAIS is based on the improved LAILX method account for the clumping index and acquire the actual LAI. A significant difference-test showed that there is no significant difference between the two kind of measurements (*p* = 0.47) according to the paired data from different corns and different sites. The LAIS has been proved effective for LAI measurement through the validation of LAI from LAILLW. The actual LAI is defined strictly as the full vegetation LAI (including stalks) [20].

### 4.3. Data Analysis

#### 4.3.1. Comparison of LAI in Different Sites

**Figure 13:**
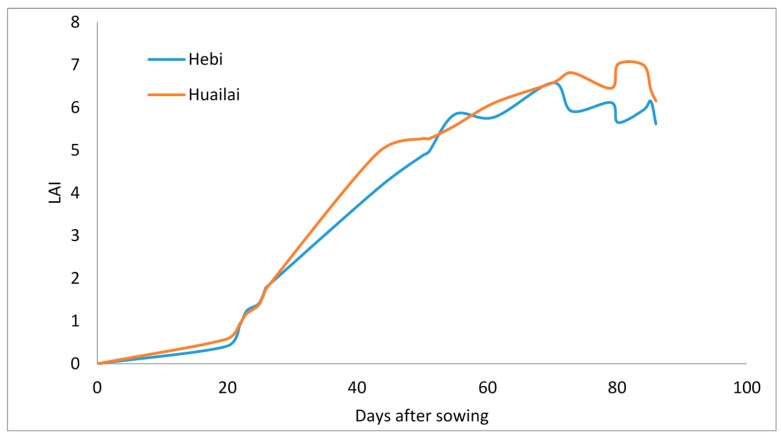
Comparison of wheat LAI change between Hebi and Huailai.

The automatically extracted LAI changes with the sowing time. Using wheat as an example, its LAI increased most quickly between 20 days to 50 days after sowing. The rate of increase for LAI dropped between 50 days to 70 days after sowing. The value of LAI decreased in volatility after 70 days after sowing. The law of LAI changes with days after sowing is basically same in Hebi and in Huailai (Figure 13).

#### 4.3.2. Comparison of LAI between Corn and Cotton

**Figure 14:**
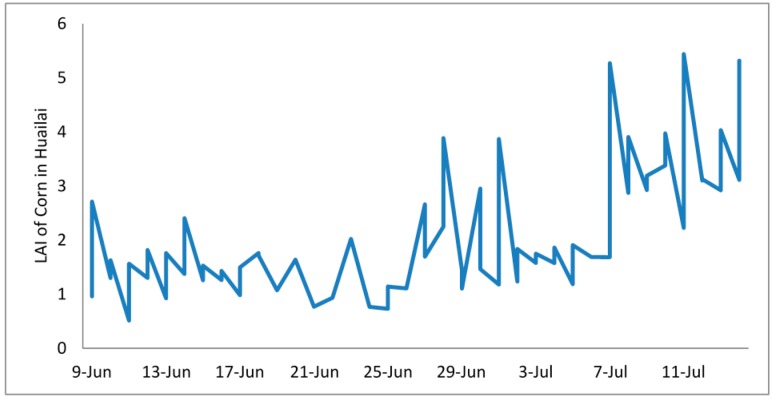
The change of corn LAI in Huailai.

**Figure 15:**
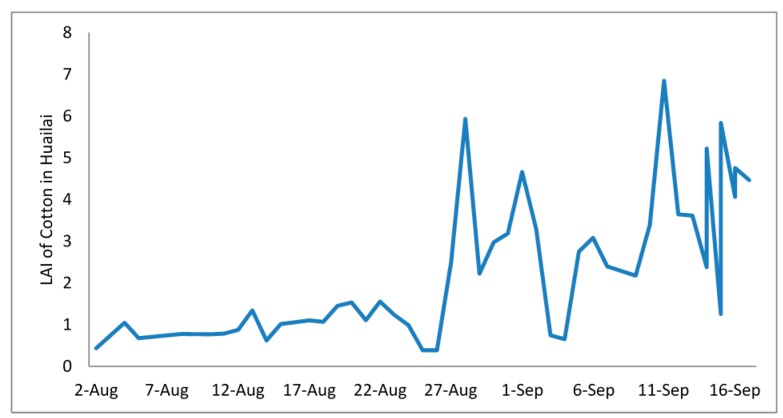
The change of cotton LAI in Huailai.

The automatically extracted LAI changes with the different crops after sowing. Using corn and cotton as examples, the changes of their LAI are quite different (Figure 14 and Figure 15).

### 4.4. Time Delay

The LAIS uses a channelized and lined 32-bit RSIC processor to collect the images. The operating frequency of this processor is 300 MHz, which is negligible. Therefore, the time delay is primarily caused by network delay and the delay due to the coding and compression of the images. The network time delay of a 3G TCP/IP network is typically from tens to hundreds of milliseconds. Because the size of a digital photo can approach approximately 10 M and because the bandwidth of a 3G network is limited, the photos are prestored on DDR2 memory and then uploaded through the 3G network. At a speed of 200 Kbp/S, the network requires 52 s to transmit a 10 M photo.

### 4.5. Error Rate

The system consumes the most energy while transmitting data. Because ejected electric currents are pulses, the power source has to provide transient currents of at least 2 A, otherwise the module will restart due to insufficient voltage, and the system will have average electric currents of no more than 500 mA while transmitting data. The energy consumption can reach a maximum of 3 W when the system begins photographing, and the energy consumption is only 1.8 mW in power-saving mode.

## 5. Discussions and Conclusions

### 5.1. Discussion

The LAIS is designed to carry out long-term and continuous monitoring of crop growth parameters, such as the LAI. It can directly provide real-time measurement data for small scale applications such as farm management, or work together with remote sensing techniques as a synchronous and independent validation/calibration data source. This paper introduces LAIS in its hardware and software systems, emphasizing its capacity and accuracy respectively.

As to the capacity, the LAIS automatically acquires high quality digital photos with a camera placed above the top of crop canopy, and instantaneously transfers the photos to the data center where extensive analysis can be carried out. Currently the photo analysis involves classification and LAI/CI estimation. New functions, such as nutrient levels, crop height, crop disease identification and visual inspection by experts, will be developed in future; and the current algorithms and hardware can also be upgraded according to the latest theoretic advances in literature and industrial innovations.

As to the accuracy, the LAIS has adopted the widely accepted Lang and Xiang method for the estimation of LAI and CI, and made improvement to the method with respect to the size of partition unit. As has been practically tested and validated with direct LAI measurement, the algorithm is robust and has acceptable accuracy. As has been pointed out in the review literature [7], it is still a challenge to accurate retrieve LAI with indirect methods. Some of the scientific problems are not treated in this preliminary study. For example, the representativeness of the FOV of the camera to the surrounding field cannot be judged by any software, but must be considered when installing the LAIS, and defining the region of interest in the configuration file. Another important aspect this study didn’t cover is the leaf angle distribution. Currently we only assume the average leaf angle can be predefined for each crop species. The method to extract leaf angle from canopy photo is the next goal of our research.

Although the LAIS has only been tested in limited circumstances and with validation data, its practical value and great potential can be foreseen: with an adequate deployment of LAIS, the growth status of crops in a region can be monitored nearly in real-time. Furthermore, combining LAIS measurement with quantitative remote sensing, the crop management and yield prediction accuracy can be enhanced. To achieve this goal, more scientific researches, as well as practical considerations and implementations, need to be invested.

### 5.2. Conclusions

The leaf area index sensor (LAIS) can acquire photos of the long-term continuous growth of crops and can collect parameters, such as the LAI, via improved methods of calculation. The LAIS has the following advantages:

(1) The software and hardware were researched and developed independently, which not only ensures a stable, expandable and reliable system but also reduces the cost.
(2) The LAIS achieves continuous automatic observation of crop conditions. The hardware design ensures reasonable hardware settings, a strong system in terms of anti-jamming, and greatly reduces the power consumption. The hardware is also capable of remote upgrades and customizing settings, and set the foundation for field deployment.
(3) The LAI estimations from photos acquired by LAIS are stable and have reasonable accuracy. It provides a more reliable and convenient way than traditional field surveys to monitor the LAI dynamics or collect synchronous validation/calibration data for remote sensing applications.

Currently, LAIS is primarily used to acquire LAI from crops including corn and wheat. In the future, more crops and more parameters can be acquired based on the collection and analysis of digital photos, including the growth rate, nutrient levels, diseases and pests. After further upgrades of the software and hardware, remote diagnosis will be realized, and significant disasters with the potential to jeopardize agricultural production will be noticed immediately.

## References

[1] Guerif G., Duke C. (1998). Calibration of the SUCROS emergence and early growth module for sugar beet using optical remote sensing data assimilation. Eur. J. Agron..

[2] Canisius F., Fernandes R., Chen J. (2010). Comparison and evaluation of Medium Resolution Imaging Spectrometer leaf area index products across a range of land use. Rem. Sens. Environ..

[3] Yao Y., Fan W., Liu Q., Li L., Tao X.X., Xiao Z., Liu Q. (2010). Improved harvesting method for corn LAI measurement in corn whole growth stages. Trans. Chin. Soc. Agric. Eng..

[4] Ross J. (1981). The Radiation Regime and Architecture of Plant Stands.

[5] Jones H.G. (1992). Plant and Microclimate.

[6] Chen J.M., Rich P.M., Gower T.S., Norman J.M., Pulmmer S. (1997). Leaf area index on boreal forests: Theory, techniques and measurements. J. Geophys. Res..

[7] Jonckheere I., Fleck S., Nackaerts K., Muysa B., Coppin P., Weiss M., Baret F. (2004). Review of methods for *in situ* leaf area index determination Part I. Theories, sensors and hemispherical photography. Agric. For. Meteorol..

[8] Kucharik C.J., Norman J.M., Murdock L.M., Gower T.S. (1997). Characterizing canopy nonrandomness with a Multiband Vegetation Imager MVI. J. Geophys. Res..

[9] Liu J.G., Pattey E. (2010). Retrieval of leaf area index from top-of-canopy digital photography over agricultural crops. Agric. For. Meteorol..

[10] Bareta F., Solana B., Lopez-Lozanoa R., Ma K., Weissa M. (2010). GAI estimates of row crops from downward looking digital photos taken perpendicular to rows at 57.5° zenith angle: Theoretical considerations based on 3D. Agric. For. Meteorol..

[11] Gong P. (2007). Wireless Sensor Network as a New Ground Remote Sensing Technology for Environmental Monitoring. J. Remote Sens..

[12] Gong P., Cheng X., Li X., Wang L., Shen S. (2009). The Application of Wireless Sensor Network Technology in Ground Environment Sensing. J. Remote Sens..

[13] Li X., Cheng X., Gong P. (2011). Design and Implementation of a Wireless Sensor Network-Based Remote Water-Level Monitoring System. Sensors.

[14] Xia J. (2011). Study on Automatic Measuring of Crop Leaf Area Index with *in Situ* Wireless Sensor. Master’s Thesis.

[15] Chen J.M., Chilar J. (1995). Plant canopy gap-size analysis theory for improving optical measurements of leaf-area index. Appl. Opt..

[16] Gregory P., Asner C.A., Wessman D.S., Schimel S.A. (1998). Variability in Leaf and Litter Optical Properties: Implications for BRDF Model Inversions Using AVHRR, MODIS, and MISR. Remote Sens. Environ..

[17] Cai B., Shao X. (2007). Leaf area index petrieval based on remotely sensed data and prospect + sail model. Remote Sens. Land Resour..

[18] Jin H., Tao X., Fan W. (2007). Application of Beijing high resolution satellite data monitoring the spatial distribution of leaf area index. Progr. Nat. Sci..

[19] Lang A.R.G. (1986). Estimation of leaf area index from transmission of direct sunlight in discontinuous canopies. Agric. For. Meteorol..

[20] Tang S., Zhu Q., Sun R. (2006). Large leaf area index inversion algorithm and its validation based on the direction of the reflectivity. Progr. Nat. Sci..

